# The role of probiotics for preventing dysbiosis in periodontal disease: a randomized controlled trial

**DOI:** 10.55730/1300-0144.5798

**Published:** 2023-12-07

**Authors:** Tuğba ŞAHİN, Gülçin AKCA, Nurdan ÖZMERİÇ

**Affiliations:** 1 Division of Periodontology, Faculty of Dentistry, University, Bolu Abant İzzet Baysal University, Bolu, Turkiye; 2 Division of Microbiology, Faculty of Dentistry, Gazi University, Ankara, Turkiye; 3 Division of Periodontics, Faculty of Dentistry, Gazi University, Ankara, Turkiye; 4 Division of Periodontics, School of Dentistry, Cyprus International University, Lefkoşa, North Cyprus, Turkiye

**Keywords:** DNA sequencing analysis, dysbiosis, kefir, periodontitis, probiotics

## Abstract

**Background/aim:**

Scaling and root planing remain inadequate in periodontitis treatment caused by dysbiotic microbial dental plaque. The aim of this clinical trial is to evaluate the effects of probiotics and kefir consumption in initial periodontal therapy (IPT) on oral microbiota composition and treatment outcomes in patients with periodontitis.

**Materials and methods:**

The study was carried out in the Gazi University Department of Periodontology, including a sample size of 36 individuals and utilizing a randomized controlled design. Thirty-six patients with periodontitis were randomly allocated to three groups: one receiving probiotic treatment, another receiving kefir, and a third serving as the control group. Obtaining subgingival microbial samples, we recorded plaque, gingival index, bleeding on probing, periodontal pocket depth, and clinical attachment level (periodontal clinical indices) and then performed IPT. For 14 days, patients took either probiotics, kefir, or no supplements. Data for the first and third months were collected using periodontal clinical indices. DNA sequencing was performed to detect *Tannerella forsythia*, *Porphyromonas gingivalis*, and *Treponema denticola* in subgingival plaque samples collected at baseline and three months.

**Results:**

Significant differences were observed regarding periodontal clinical indices among groups in the intragroup comparisons. Moreover, levels of *Tannerella forsythia* were significantly decreased in all groups.

**Conclusion:**

Kefir can be administered in addition to IPT, providing results similar to those observed with probiotics.

## 1. Introduction

Periodontitis, a chronic inflammatory disease, is closely linked to dysbiotic microbial dental plaque and is characterized by the gradual deterioration of the tissues that support the teeth [1]. *Porphyromonas gingivalis* (*P. gingivalis*), *Tannerella forsythia* (*T. forsythia*), and *Treponema denticola* (*T. denticola*) are considered the main pathogens causing periodontitis [2]. Scaling and root planing, known as SRP, is a highly prevalent mechanical therapy that has proven to be effective in the treatment of periodontal diseases and the prevention of their recurrences following treatment [3]. It has been suggested in several studies that scaling-alone may not be entirely effective in eliminating periodontopathogens. Furthermore, it is essential to note that periodontal pockets, which serve as reservoirs for periodontopathogens, have the potential to persist [3, 4]. Hence, it becomes crucial to evaluate the efficacy of recently produced products in effectively eradicating bacteria over a prolonged period of time [5].

Probiotics are defined as live microorganisms which, when administered in sufficient quantities, provide advantageous effects on the host organism [6]. Kefir, a fermented beverage derived from kefir grains, combines yeasts that ferment lactose with yeasts that do not. These yeasts have a mutually beneficial relationship with bacteria that produce lactic and acetic acids [7]. Lactic acid bacteria (LAB) represent the prevailing microorganisms employed as probiotics and are categorized into six distinct groups, namely *Lactobacillus*, *Bifidobacterium*, *Enterococcus*, *Streptococcus*, *Leuconostoc*, and *Pediococcus* [8].

Studies indicate that LAB potentially exerts influence within the oral cavity, employing intricate mechanisms that encompass the release of diverse antimicrobial agents, including lactic acid, acetic acid, hydrogen peroxide, carbon dioxide, and bacteriocin. Furthermore, it is worth noting that there is a possibility of the presence of probiotic bacteria within the oral microflora. These bacteria are believed to play a significant role within the intricate ecosystem of dental plaque, as well as in the creation and progression of oral biofilms [9]. Therefore, several studies have evaluated the use of probiotic products containing Streptococcus, *Lactobacillus*, or *Bifidobacterium* [10] in the treatment of dental caries and periodontal treatment [11]. These studies suggest that the administration of probiotics may yield favorable outcomes in the context of periodontal treatment. Furthermore, a number of research are consistent with the idea that kefir has the capacity to reduce the quantity of cariogenic bacteria [12, 13]. Nevertheless, the efficacy of kefir in the management of periodontitis remains yet to be explored, as no scientific investigations have been conducted to assess its potential therapeutic benefits in this particular context.

The aim of this study is to assess the effect of probiotic consumption in chewable tablets and kefir, when combined with initial periodontal treatment (IPT), on the composition of oral microbiota. Additionally, the study aims to evaluate the enhancement of gingival health in patients.

## 2. Materials and methods

### 2.1. Study design

This randomized controlled trial was conducted in compliance with the guidelines of the Consolidated Standards of Reporting Trials (CONSORT) and the Declaration of Helsinki, revised in 2013. This clinical study was registered at ClinicalTrials.gov (NCT05211219, 03/2020-18).

The study was conducted at a single center, specifically Gazi University Faculty of Dentistry, in the Department of Periodontology. It included patients admitted with complaints of stage 1 and stage 2 periodontitis, diagnosed through clinical and radiographical examinations. The age range of the participants was 18–70 years. The participants were informed about the nature of the proposed treatment, its risks, and benefits, and signed the consent form.

### 2.2. Eligibility criteri

The inclusion criteria included systemically healthy individuals with untreated periodontitis exhibiting periodontal pocket depths ranging from 4 to 6 mm. Exclusion criteria, on the other hand, were as follows: antibiotic use within six months, breastfeeding or pregnancy, acute oral lesions, systemic diseases, smoking or not quitting smoking by the year before the study, use of probiotic tablets/capsules, and consumption of probiotic food more than 3–4 times a week.

### 2.3. Data collection

Plaque [14], gingival index [15], bleeding on probing [16], periodontal pocket depth, and clinical attachment level were evaluated. The patients were then randomly divided into three groups: probiotic + IPT (group 1), kefir + IPT (group 2) and IPT (group 3). After the first visit, clinical index records (T0), the clinician (T. Ş.) made the periodontal diagnosis and performed subgingival microbial sampling. During the same visit, patients underwent SRP and simultaneously received probiotics (Probest Defense Abdi İbrahim^TM^, İstanbul, Türkiye) either as a chewable tablet or kefir (Atatürk Orman Çiftliği, Ankara, Türkiye) (250 mL) once a day for 14 days, depending on their treatment groups. Patients were asked to consume kefir without rinsing in the mouth, while those in the control group were asked not to consume additional food supplements. After consuming kefir or probiotics between 9.00 a.m. and 10.00 a.m. following breakfast, the patients were instructed not to eat or drink for two hours. To maintain the integrity of the study results, patients were explicitly instructed to restrict their consumption of fermented foods and probiotics to a maximum of three times per week. The indicators underwent an assessment on two occasions: firstly, at the beginning of the trial (T1), and subsequently, three months afterward (T2). When periodontitis treatment was concluded, there was a plaque index of less than 10%, and probing of periodontal pockets showed no bleeding.

### 2.4. Microbiological analysis

The subgingival microbial dental plaque samples were obtained at the first and third months. The clinician gently removed the subgingival microbial dental plaque from the deepest periodontal pockets of the patients using a sterile Gracey curette (Hu-Friedy, Chicago, USA). Subsequently, the levels of *T. forsythia*, *P. gingivalis*, and *T. denticola* were determined through DNA sequencing. DNA was isolated from the samples using the kit (GeneMATRIX, EurXTM, Gdansk, Poland) in line with the manufacturer’s instructions. Prior to DNA sequencing readout, the 16S V3 and V4 regions in each sample were amplified using PCR during amplicon library preparation. For the DNA purification, primer dimers and free primers were removed using magnetic beads (AMPure XP, Beckman Coulter, California, USA). This method was used to determine the genes by searching the sequence in terms of similarities and protein-coding potential and mutations in the genes, and also by comparing the sequences of the identified bacteria with those registered in Genbank (NCBI, GenBank, Bethesda, USA).

### 2.5. Outcome variables

#### 2.5.1. Primary outcome variables

The primary outcomes encompassed various aspects of periodontal assessment, including periodontal probing depth, bleeding on probing, clinical attachment level, plaque index [14], and microbial parameters. The clinician conducted all examinations employing a UNC-15 periodontal probe (Hu-Friedy based in Chicago, USA). The PI was noted according to Silness and Loe (1964) [14]. The clinical attachment level (CAL) was assessed by measuring the distance between the cementum-enamel junction and the gingival margin in six regions.

#### 2.5.2. Secondary outcome variables

The secondary outcome was the gingival index, which was assessed in accordance with Loe and Silness (1963) [16]. The assessment involved examining six distinct sites on each tooth: mesiobuccal, mid-buccal, distobuccal, mesiolingual, mid-lingual, and distolingual regions.

### 2.6. Sample size

A total sample size of 36 (12 for each group) was determined to be necessary to ascertain a minimum effect size of 1.20 (known as Cohen’s d) between any two groups while maintaining a power of 80% at the 5% significance level. The value of 1.20 was obtained from the clinical experiments. The sample size estimation was conducted utilizing G*Power (Franz Faul, Universität Kiel, Kiel, Germany) version 3.0.10. The allocation of groups was achieved through the sealed envelope technique via a randomization process (n=24).

### 2.7. Statistical analysis

Data analysis was done in IBM SPSS Statistics ver. 25 (IBM Corporation, Armonk, NY, USA) package program. The Kolmogorov-Smirnov and Levene’s tests were respectively used to investigate whether the parametric test assumptions of normal distribution and homogeneity of variances were met. Quantitative data were shown as median (25th–75th) percentiles. Whether the differences in continuous variables (i.e. plaque index, gingival index, pocket depth, or probing across groups based on the levels of bleeding and attachment loss, 4–6 mm PPD, microbiological measurements) among study groups were statistically significant or not was evaluated by Kruskal-Wallis test. When the p-values from Kruskal-Wallis test were statistically significant, the Dunn-Bonferroni test was used to determine which group differs from which others. While the differences in the number of growing microorganisms (i.e. *T. forsythia*, *P. gingivalis* and *T. denticola*) between pre- and posttreatment within each group were compared by Wilcoxon signed rank test, otherwise the Friedman test was applied for the comparisons among baseline, the 1st and the 3rd month in terms of clinical periodontal measurements (i.e. PI, GI, PPD, BOP, and CAL). When the p-values obtained from the Friedman test were statistically significant, the Dunn-Bonferroni test was performed to determine which follow-up period caused the difference. A p-value less than 0.05 was considered statistically significant. However, for all possible multiple comparisons, the Bonferroni adjustment was applied to control type I error.

## 3. Results

**Figure 1:**
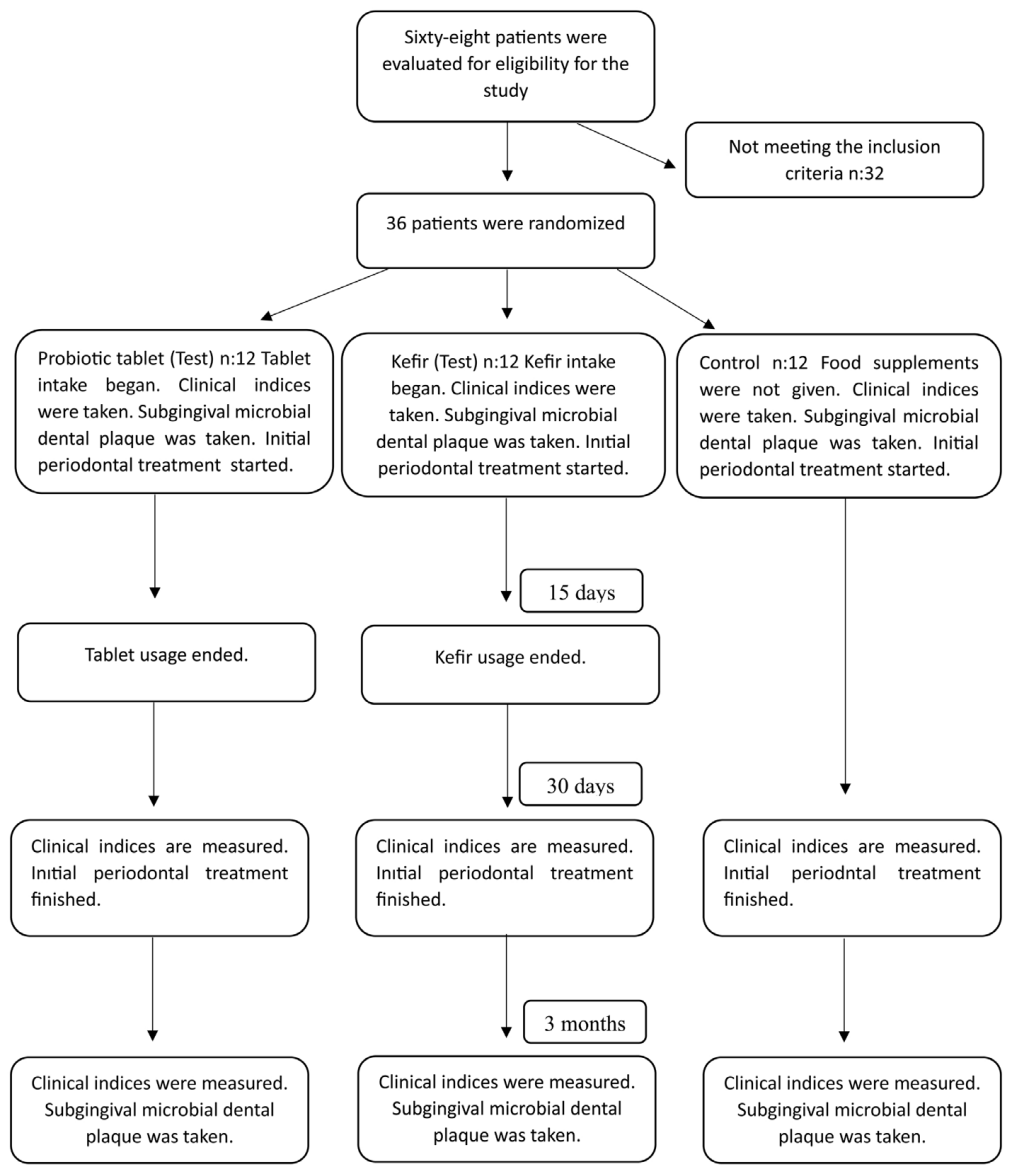
Flow chart.

All patients complied with the treatment processes; none were lost to follow-up (Figure 1).

The examination of demographic characteristics across the three groups revealed no statistically significant differences (p = 0.528 and p = 0.429).

**Figure 2:**
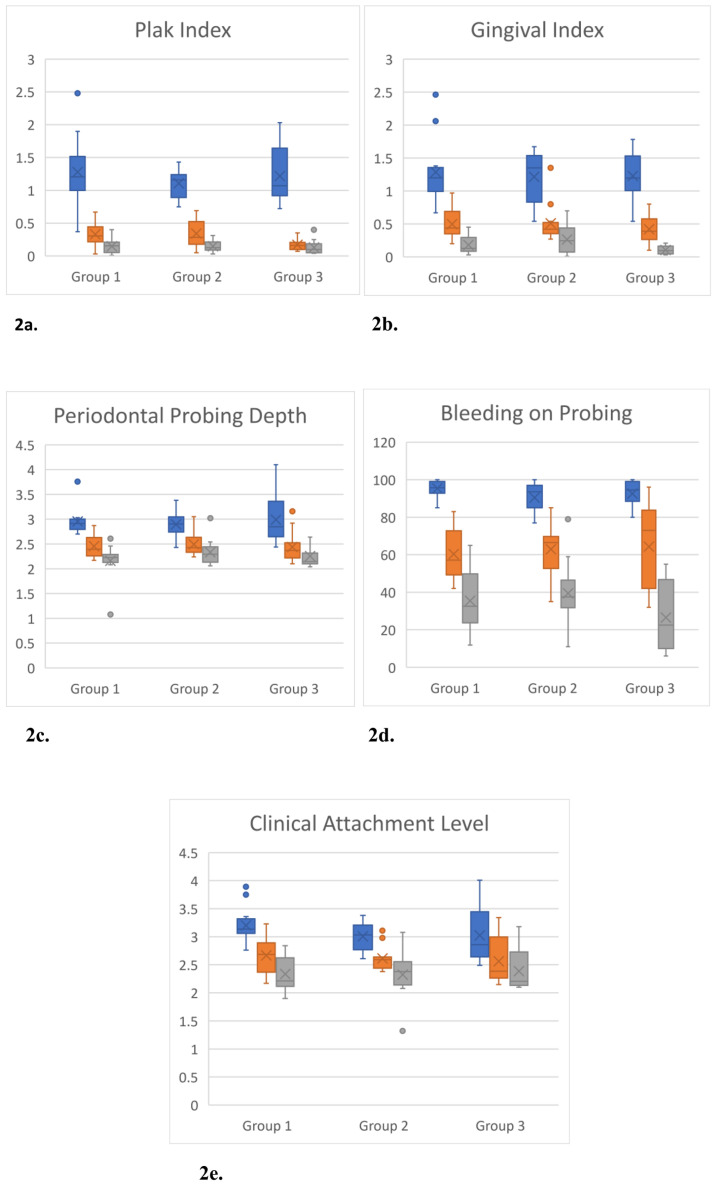
Distribution of periodontal indices.

There was no statistically significant difference in baseline (T0) plaque index, gingival index, pocket depth, or bleeding on probing across groups based on the levels of bleeding and attachment loss (p = 0.680, p = 0.906, p = 0.960, p = 0.279, and p = 0.270, respectively). In each of the three experimental cohorts, a significant reduction in the levels of all clinical indices was observed at T2 in comparison to baseline measurements at T0 (p < 0.001) (Figures 2a–2e).

Within the examined cohorts, specifically T1 in relation to T0, T2 in relation to T0, and T2 in relation to T1, no significant difference was observed in any of the clinical parameters assessed (p > 0.0167) (Table 1).

A statistically significant difference was observed in the 4–6 PPD (pocket probing depth) measurements across the follow-up periods in group 1, group 2, and group 3 (p < 0.001), which was due to a reduction in PPD at T2 in comparison to T0 (p < 0.001). The analysis revealed no statistically significant difference between the groups in relation to the levels of PPD measurements observed at both T0 and T1, as well as T1 and T2, as determined through Bonferroni correction (p = 0.043 in G-1, p = 0.024 in G-2 and G-3 for T0 vs. T1 and p = 0.043 in G-1, p = 0.124 in G-2 and G-3 for T1 vs. T2). After applying the Bonferroni correction, no statistically significant difference was observed among study groups in terms of the differences observed in the measurements of PPD in T1 vs. T0, T2 vs. T0, and T2 vs. T1 (p = 0.096, p = 0.265, and p = 0.522, respectively) (Table 2).

The levels of *T. forsythia* were significantly reduced after the treatment compared to pretreatment among all bacteria detected in the three groups (p = 0.006, p = 0.008, and p = 0.012, respectively). However, the reduction in the levels of *T. forsythia* after the treatment compared to pretreatment in all bacteria was statistically similar between groups (p = 0.623). Moreover, there was a reduction in the level of *P. gingivalis* and *T. denticola* between treatment and pretreatment in all bacteria detected in each group. However, these changes were not found to be statistically significant after the Bonferroni correction method (p = 0.062 in G-1, p = 0.123 in G-2, p = 0.017 in G-3 for *P. gingivalis* and p = 0.017 in G-1, p = 0.026 in G-2, p = 0.060 in G-3 for *T. denticola*). Of all the bacteria, the decrease in the level of *P. gingivalis* and *T. denticola* after treatment compared to pretreatment was statistically similar between the three groups (p = 0.688 and p = 0.694) (Table 3).

The overall number of bacteria found before treatment in each group and the amounts of *T. forsythia*, *P. gingivalis*, and *T. denticola* were not significantly different across the groups (p = 0.320, p = 0.073, and p = 0.506, respectively).

This randomized controlled trial evaluated the impact of probiotic bacteria on initial periodontal therapy in individuals diagnosed with periodontitis. The study analyzed various clinical, immunological, and microbiological parameters to assess the effects of probiotic intervention. The findings indicate that the utilization of probiotic therapy, specifically kefir and probiotic tablets, in conjunction with scaling and root planing (SRP), yields comparable outcomes to those observed in the control group during assessments conducted at T1 and T2.

## 4. Discussion

The presence of bacteria in the periodontal area extends into the deeper layers of tissues and the surrounding periodontium [17]. The SRP procedure is considered the primary gold standard treatment for periodontitis. It is important to note that scaling alone remains insufficient in effectively reducing the presence of subgingival microbiota [3, 18, 19]. Therefore, it is recommended to administer antimicrobial treatment in conjunction with scaling and root planing [18]. In addition to antimicrobial therapy, it is also possible to consider the implementation of antibiotics [20], photodynamic [21], and probiotic therapies [22] as potential treatment options. The oral cavity stands out as the initial segment of the gastrointestinal system. According to some studies, it has been suggested that LAB may play a potential role within the oral cavity [10] through mechanisms involving the release of diverse antimicrobial substances, such as lactic acid, acetic acid, hydrogen peroxide, carbon dioxide, and bacteriocin [23]. It can be concluded that probiotics may offer supplementary advantages in addition to manual therapy [24].

The clinical indices provide information on the pathogenesis and progression of periodontal disease [25]. In a study by Morales et al. (2018), a probiotic tablet [*L. rhamnosus* SP1 (*Lactobacillus rhamnosus* SP1)] was administered once a day for three months, yielding a significant decrease in PI in all groups (probiotic, antibiotic, and control) [26]. However, the groups had no significant differences in BOP, PPD, CAL, and microbial dental plaque accumulation. The PPD consistently decreased throughout the study in the probiotic group, while clinical attachment gain was observed between the third and ninth months. A study by Ikram et al. points out a statistically significant difference in the within-group (both probiotic and antibiotic groups) analysis of periodontal parameters, such as PI and BOP, with a decrease in proinflammatory cytokines; however, the comparison between the groups failed to show statistical significance in clinical periodontal parameters [11]. The present study observed a significant decrease in intragroup results in all clinical indices between baseline and the third month in all groups. However, based on a between-group comparison, no significant difference was observed. Despite the antimicrobial effects of probiotic bacteria (*L. rhamnosus)* on gram-negative periodontopathogens, Morales et al. observed an increase in the total bacteria and *P. gingivalis, A. actinomycetemcomitans*, and *T. forsythia* amounts among all groups.

There were no significant differences at the 3rd, 6th, and 9th month follow-ups [26]. In the present study, no significant difference was observed between the groups in terms of *P. gingivalis*, *T. denticola* and *T. forsythia* levels. According to between-group comparisons, no statistically significant difference was observed in the numbers of *P. gingivalis* and *T. denticola* among all groups.

*T. forsythia* and *T. denticola* are associated with the severity of periodontitis [27]. *T. forsythia* is a pathogenic organism that can synergize with the inflammatory role of other periodontopathogens [28]. Laleman et al. observed that *T. forsythia* decreased significantly in the supragingival plaque in one month [29]. Moreover, Mayanagi et al. also found that the number of *T. forsythia* in the test group decreased statistically significantly within four weeks [30].

There are no studies on kefir consumption in patients with periodontal disease; thus, it would be interesting to examine the effects of kefir on bacteria in periodontitis. Cogulu et al. found statistically significant reductions in the levels of *S. mutans* and *Lactobacillus* in the saliva of patients with cavities who consumed 200 mL of kefir [17]. Alp et al. examined the patients in probiotic and kefir groups, concluding a decrease in the levels of *S. mutans* and *Lactobacillus* in patients who consumed 200 mL of kefir or used probiotic paste. However, the difference between the groups was not statistically significant [16].

A significant limitation of the present study was the small sample size and short-term follow-up period. Thus, further studies involving larger sample size and longer- or shorter-term follow-ups are needed to ascertain a more valid difference between the two adjunctive therapeutic agents. Additionally, kefir should be compared with probiotics in non-tablets. Therefore, further studies are needed to compare the efficacy of these adjunctive therapies against red-complex bacteria and evaluate their effects on clinical indices.

This study highlighted that although SRP is the gold standard treatment for periodontitis, additional probiotics are an excellent alternative. For the first time, this study evaluated the efficacy of kefir, a probiotic beverage. Kefir improved clinical and microbiological outcomes in patients with periodontitis, similar to those observed with other probiotics. The study also indicated the importance of SRP in the treatment of periodontitis. Additionally, the positive effects of kefir on the levels of *T. Forsythia* could promote more research on using kefir in different designs.

## Figures and Tables

**Table 1:** Comparisons between groups for changes in clinical measures at any two follow-up times.

	G-1	G-2	G-3	p-value†
**PI**				
**1 month–baseline**	−1.03 (−1.26 to −0.57)	−0.81 (−0.99 to −0.57)	−0.99 (−1.33 to −0.77)	0.281
**3 month –baseline**	−1.13 (−1.23 to −0.86)	−1.00 (−1.13 to −0.70)	−1.01 (−1.31 to −0.78)	0.483
**3 month –1 month**	−0.14 (−0.41 to 0.02)	−0.19 (−0.31 to −0.02)	−0.03 (−0.09 to 0.00)	0.129
**GI**				
**1 month–baseline**	−0.78 (−1.00 to −0.33)	−0.86 (−0.97 to −0.34)	−0.82 (−1.08 to −0.60)	0.693
**3 month–baseline**	−1.06 (−1.31 to −0.58)	−1.14 (−1.29 to −0.42)	−1.15 (−1.36 to −0.94)	0.770
**3 month –1 month**	−0.29 (−0.52 to −0.18)	−0.28 (−0.39 to −0.05)	−0.26 (−0.43 to −0.21)	0.789
**PPD**				
**1 month–baseline**	−0.51 (−0.60 to −0.32)	−0.46 (−0.61 to −0.26)	−0.49 (−0.64 to −0.36)	0.762
**3 month –baseline**	−0.68 (−0.87 to −0.55)	−0.57 (−0.80 to −0.40)	−0.70 (−0.90 to −0.47)	0.442
**3 month–1 month**	−0.25 (−0.31 to −0.18)	−0.18 (−0.20 to −0.12)	−0.18 (−0.28 to −0.12)	0.122
**BOP**				
**1 month–baseline**	−38.13 (−45.75 to −27.00)	−28.00 (−32.50 to −25.93)	−27.00 (−44.50 to −12.25)	0.322
**3 month–baseline**	−63.00 (−71.50 to −48.25)	−53.00 (−63.75 to −35.75)	−69.00 (−78.00 to −50.25)	0.136
**3 month –1 month**	−24.50 (−39.00 to −8.50)	−26.00 (−33.25 to −13.98)	−34.50 (−40.75 to −26.00)	0.115
**CAL**				
**1 month–baseline**	−0.57 (−0.71 to −0.34)	−0.47 (−0.60 to −0.19)	−0.41 (−0.60 to −0.35)	0.390
**3 month–baseline**	−0.89 (−1.20 to −0.56)	−0.60 (−0.85 to −0.40)	−0.66 (−0.81 to −0.45)	0.135
**3 month–1 month**	−0.24 (−0.39 to −0.15)	−0.18 (−0.40 to −0.05)	−0.16 (−0.24 to −0.10)	0.422

Descriptive statistics were expressed as median (25th percentile–75th percentile),

† Kruskal-Wallis test,

Bonferroni correction for p < 0.0167, and the results were considered statistically significant.

**Table 2:** According to the groups and follow-up times, 4–6 mm PPD.

	G-1	G-2	G-3	p-value†¶
**PPD**				
**Baseline**	4.56 (4.41–4.73)^a^	4.53 (4.37–4.75)^a^	4.59 (4.44–4.79)^a^	0.804
**1 month**	2.89 (2.70–3.28)	3.19 (2.87–3.66)	3.00 (2.76–3.37)	n/a
**3 month**	2.49 (2.33–2.78)^a^	2.73 (2.31–3.21)^a^	2.51 (2.27–3.01)^a^	n/a
**p-value** ‡¶	**<0.001**	**<0.001**	**<0.001**	
**Variations**				
**1 month–baseline**	−1.72 (−1.87 to −1.31)	−1.27 (−1.51 to −1.20)	−1.51 (−1.88 to −1.36)	0.096
**3 month– baseline**	−2.01 (−2.17 to −1.81)	−1.82 (−2.03 to −1.51)	−1.98 (−2.34 to −1.70)	0.265
**3 month– 1 month**	−0.51 (−0.63 to −0.23)	−0.53 (−0.59 to −0.42)	−0.40 (−0.52 to −0.34)	0.522

Descriptive statistics were expressed as median (25th percentile–75th percentile),

† Kruskal-Wallis test,

‡ Friedman test,

¶ according to Bonferroni correction, the results were considered statistically significant for p < 0.0167.

n/a: No evaluation was made,

a the difference between baseline and third month was statistically significant (p < 0.001).

**Table 3:** The incidence of pre-treatment and post-treatment bacterial counts among all bacteria in the groups.

	Before treatment	After treatment	p-value†	Variation	p-value ^‡^
***T. forsythia***					0.623
**G-1**	0.73 (0.53–1.44)	0.05 (0.01–0.29)	**0.006**	−126.6 (−195.2 to −91.7)	
**G-2**	1.08 (0.58–1.71)	0.41 (0.12–0.63)	**0.008**	−105.9 (−151.2 to −58.0)	
**G-3**	1.34 (0.23–2.86)	0.16 (0.01–0.43)	**0.012**	−127.0 (−198.3 to −7.5)	
***P. gingivalis***					0.688
**G-1**	3.25 (0.33–6.62)	0.19 (0.02–2.10)	0.062	−156.1 (−198.0 to 18.2)	
**G-2**	0.99 (0.00–2.36)	0.16 (0.00–0.86)	0.123	−97.6 (−169.9 to −47.2)	
**G-3**	4.53 (0.80–10.93)	1.17 (0.01–3.50)	0.017	−128.3 (−192.8 to −31.2)	
***T. denticola***					0.694
**G-1**	1.17 (0.56–2.48)	0.53 (0.04–0.74)	0.017	−106.7 (−178.1 to −41.2)	
**G-2**	1.56 (1.31–2.04)	0.76 (0.48–1.13)	0.026	−78.3 (−120.6 to −26.4)	
**G-3**	1.31 (1.01–2.64)	0.35 (0.13–0.87)	0.060	−69.3 (−171.7 to −50.8)	

Descriptive statistics were expressed as median (25th percentile–75th percentile),

† Comparisons made in pretreatment and posttreatment bacterial rates within groups,

Wilcoxon signed rank test, Bonferroni correction for p < 0.0167.
